# Mechanical Properties of Elastomeric Impression Materials: An In Vitro Comparison

**DOI:** 10.1155/2015/428286

**Published:** 2015-11-26

**Authors:** Dino Re, Francesco De Angelis, Gabriele Augusti, Davide Augusti, Sergio Caputi, Maurizio D'Amario, Camillo D'Arcangelo

**Affiliations:** ^1^Department of Oral Rehabilitation, Istituto Stomatologico Italiano, University of Milan, 20122 Milan, Italy; ^2^Department of Medical, Oral and Biotechnological Sciences, University of Chieti, 66100 Chieti, Italy; ^3^Department of Life, Health and Environmental Sciences, University of L'Aquila, 67100 L'Aquila, Italy

## Abstract

*Purpose.* Although new elastomeric impression materials have been introduced into the market, there are still insufficient data about their mechanical features. The tensile properties of 17 hydrophilic impression materials with different consistencies were compared.* Materials and Methods.* 12 vinylpolysiloxane, 2 polyether, and 3 hybrid vinylpolyether silicone-based impression materials were tested. For each material, 10 dumbbell-shaped specimens were fabricated (*n* = 10), according to the ISO 37:2005 specifications, and loaded in tension until failure. Mean values for tensile strength, yield strength, strain at break, and strain at yield point were calculated. Data were statistically analyzed using one-way ANOVA and Tukey's tests (*α* = 0.05).* Results.* Vinylpolysiloxanes consistently showed higher tensile strength values than polyethers. Heavy-body materials showed higher tensile strength than the light bodies from the same manufacturer. Among the light bodies, the highest yield strength was achieved by the hybrid vinylpolyether silicone (2.70 MPa). Polyethers showed the lowest tensile (1.44 MPa) and yield (0.94 MPa) strengths, regardless of the viscosity.* Conclusion.* The choice of an impression material should be based on the specific physical behavior of the elastomer. The light-body vinylpolyether silicone showed high tensile strength, yield strength, and adequate strain at yield/brake; those features might help to reduce tearing phenomena in the thin interproximal and crevicular areas.

## 1. Introduction

The success rate of prosthetic tasks relies on different factors. Adequate clinical protocols [1, 2] based on careful tooth preparations and standardized luting or cementation procedures [3, 4] proved to be crucial. Similarly, the dimensional accuracy and a reliable detailed reproduction of both impressions and corresponding models from which a restoration can be manufactured in the laboratory appear mandatory [5]. The ideal impression material should exhibit adequate mechanical properties to withstand stresses under various clinical scenarios. Elastomeric impression materials offer high elastic recovery and acceptable flexibility on removal of the impression from the mouth [6]. Recently, new elastomeric impression materials have been introduced, with the claim of very high elastic recovery and high tear and tensile strengths. Vinylpolysiloxanes (VPSs) (addition silicones) have a moderately low-molecular-weight silicone that contains silane groups. Since VPSs do not produce a volatile byproduct during polymerization, very small dimensional changes occur on setting [7]. VPS are intrinsically hydrophobic in nature, which can result in voids at the margin of the tooth preparation in the impression and bubbles in gypsum casts. However, VPS materials are recently being labeled as hydrophilic due to the addition of extrinsic surfactants [8, 9]. Polyethers (PE) are composed of a moderately low-molecular-weight polyether, a silica filler, and a plasticizer. Dimensional stability and wettability (resulting in minimal voids and detailed reproduction of intraoral structures) are the main features of PE materials [10, 11]. On the other hand, a difficulty of removing impressions made of polyether from the mouth, and also an increased risk of die breakage, could be associated with the higher rigidity of these materials when compared to VPS [11]. Recently, vinylpolyether silicone (VPES) products were commercially introduced. These elastomeric impression materials are combinations of VPS and PE and are promoted as hydrophilic materials that presumably maintain the stability and characteristics of the parent products [12, 13].

Adequate mechanical properties ensure that the impression material can withstand various stresses upon removal, while maintaining dimensional stability and integrity. The tear of elastomeric materials is a mechanical rupture process initiated and propagated at a site of high stress concentration caused by cut, defect, or localized deformation.* Tear*,* tensile*, and* yield strengths* are important properties for impression materials; they have been investigated by several studies [6, 9, 14, 15]. Lu et al. have found a lower tensile strength of a soft polyether (Impregum, 3M ESPE) compared to two hydrophilic addition silicones (Imprint II, 3M ESPE and Flexitime, Heraeus); the authors also reported higher tear properties and tensile strength of heavy-body materials than light viscosities [9]. Chai et al.—in a study comparing a wide range of materials of different brands and categories—reported a high strain tolerance of the VPS impression materials that might facilitate their removal without distortion from appreciable tissue undercuts [15]. Moderate rigidity of polyether was also recognized [15]. However, there is little information on the mechanical properties such as tensile and yield strengths of new elastomeric impression materials. Knowledge of these clinically relevant mechanical properties facilitates the selection of impression materials in various clinical situations.

The purpose of this study was to compare tensile properties (tensile strength at break, yield strength, ultimate strain at break, and strain at yield point) of 17 hydrophilic elastomeric impression materials. Materials with different consistencies (heavy-, medium-, and light-body) were investigated. The tested null hypothesis was the fact that there would be no significant differences in mechanical properties among these impression materials.

## 2. Materials and Methods

Tensile strength at break (TSb), yield strength (YS), ultimate strain at break (USb), and strain at yield point (Sy) of seventeen commercially available elastomeric impression materials with heavy- (HB), medium- (MB), or light-body (LB) consistencies were evaluated in this study. The complete list of the materials employed is summarized in Table 1 and included 12 VPSs, 2 PEs, and 3 VPESs.

**Figure 1:**
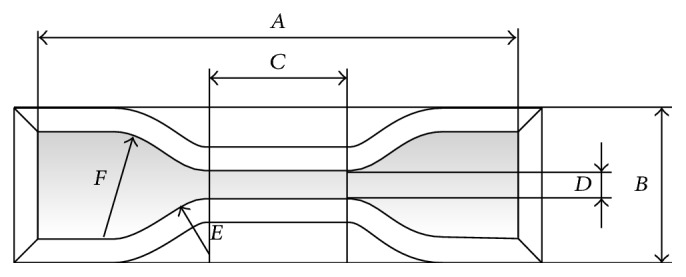
Shape of dumbbell test specimens produced according to type 1 of the ISO 37:2005 specifications and/or type C of ASTM.D412 specifications. The actual extent of the dimensions indicated by uppercase letters is specified in Table 2.

For each impression material, 10 dumbbell-shaped specimens were fabricated (*n* = 10), according to the design described as type 1 and type C, respectively, within the ISO 37:2005 and within the ASTM.D412 specifications (see Figure 1 and Table 2).

**Figure 2:**
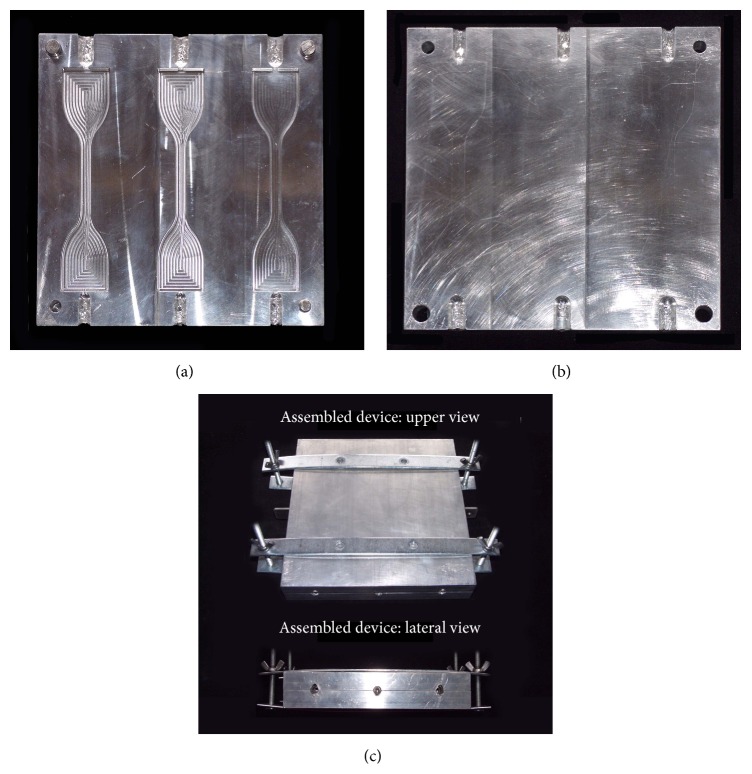
Stainless steel split mold used to produce the dumbbell test specimens: lower (a) and upper (b) plates. Once the mold was assembled (c), paths for the injection of the impression materials through lateral apertures were made available.

**Figure 3:**
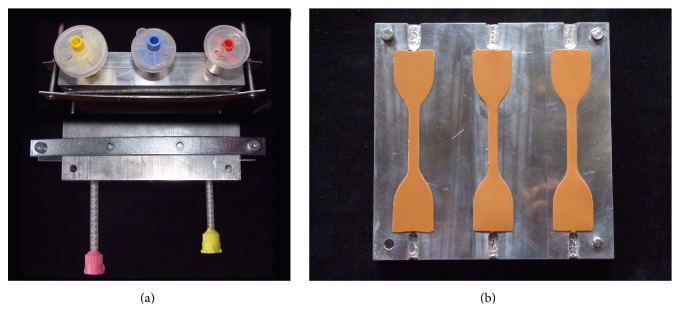
Injection of the impression materials through dedicated, commercially available tips (a) allowed the production of up to three samples at the same time (b).

For this purpose, a stainless steel split mold made out of two perfectly fitting upper and lower plates was used (Figures 2(a) and 2(b)). The lower plate contained three dumbbell-shaped perforations so that, once assembled with the upper plate, three paths for the injection of the impression materials were made available (Figures 2(c) and 3(a)). This allowed for the production of up to three samples at the same time (Figure 3(b)).

The specimens were prepared at standard laboratory conditions (23°C ± 1°C) by dispensing impression material from the cartridge into the already assembled steel mold, through lateral apertures specifically designed for placing the differently shaped cartridge tips. Before injection, a small amount of material was extruded and discarded to ensure proper mixing in the dispensing tip. A timer was started immediately after the impression material was first dispensed into the mold.

The upper and the lower plates of the split mold were kept assembled for the whole setting time recommended by each manufacturer and under a constant 5 Kg load. After complete setting and mold removal, any excess impression material residue was carefully trimmed away with a razor blade. Benchmarks were drawn on the specimen, 12.5 mm on either side of the center line, thus setting the test length of the dumbbell specimens at 25 mm, according to ISO 37:2005 and ASTM.D412 (Table 2). Specimen dimensions were recorded with a digital caliper (Mitutoyo, Tokyo, Japan) before testing. Three areas of each specimen narrow portion were measured and checked three times to accurately confirm their width and thickness, which were averaged to obtain a final measurement. Specimens that were not in accordance with the dimensions specified within the ISO 37:2005 (Table 2) were discarded; entirely new specimens were subsequently prepared.

**Figure 4:**
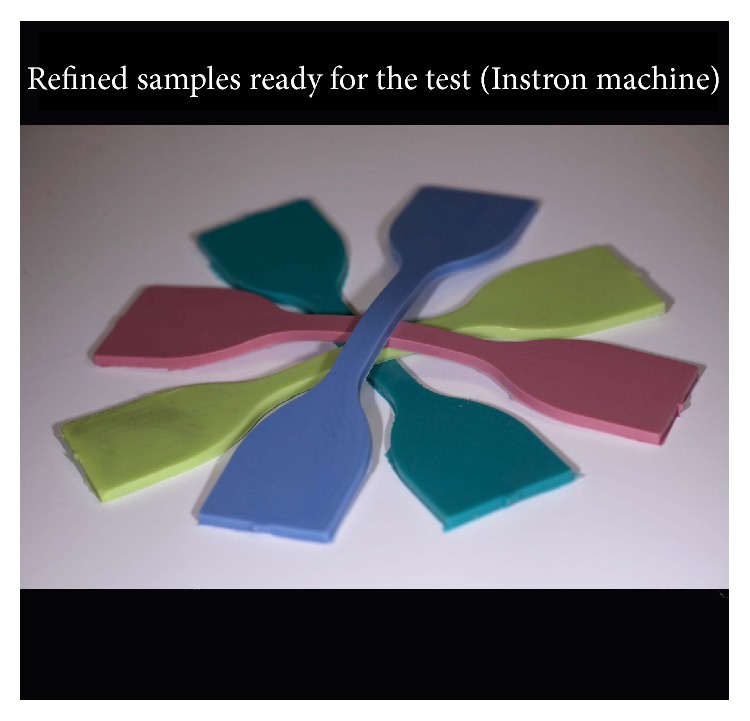
Refined dumbbell test specimens ready for the test (Instron machine).

**Figure 5:**
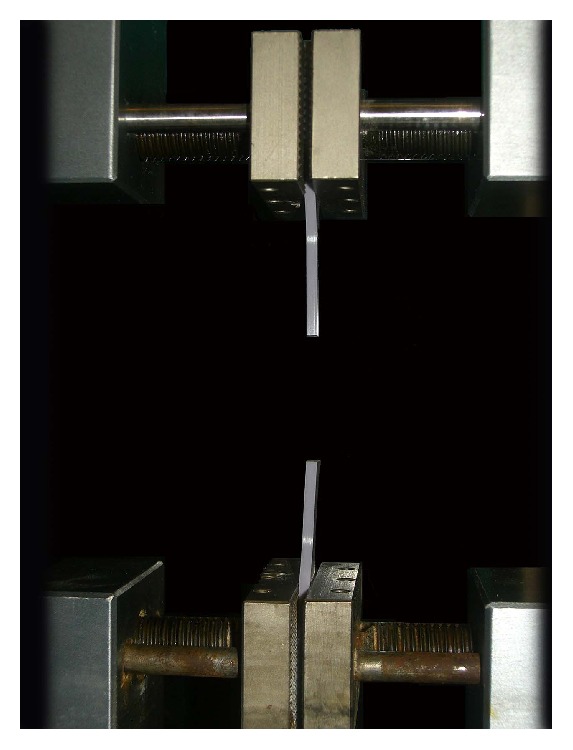
Example of a specimen loaded in tension until failure.

Immediately following preparation (Figure 4), the specimens were secured into the Instron universal testing machine (Instron Corp., Canton, MA, USA), gripping them on both sides by pneumatic clamps at the location of the previously applied benchmarks. Before the test began, the jig was adjusted so that the specimen was neither in compression nor in tension. The specimens were loaded in tension until failure (Figure 5) with a crosshead speed of 250 mm/minute. The yield point was defined according to the 0.2% offset method, by estimating a 0.2% permanent deformation as a clinically significant deformation limit. The USb (mm) and the Sy (mm) were recorded.

The TSb (MPa) was calculated using the equation

(1) $$\begin{array}{c} \text{TSb}=\frac{\text{Fb}}{W*t}, \end{array}$$

while the YS (MPa) was obtained from the force recorded at the yield point using the equation

(2) $$\begin{array}{c} \text{YS}=\frac{\text{Fy}}{W*t}, \end{array}$$

where Fb (N) is the force recorded at brake, Fy (N) is the force recorded at yield, *W* (mm) is the width of the narrow portion of the die, and *t* (mm) is the thickness of the test length.

Mean values and standard deviations for TSb, YS, USb, and Sy were calculated in each group. A normal distribution was verified for the analyzed variables before applying statistical tests. Data were subjected to a one-way analysis of variance (ANOVA) and Tukey's HSD test for multiple comparisons. The level of *α* was set at 0.05 in all tests.

## 3. Results

Mean values and standard deviations achieved for TSb, YS, USb, and Sy are shown in Table 3, which also summarizes the results of the one-way ANOVA and Tukey's tests.

VPSs consistently showed higher tensile strength at brake (TSb) values than PEs. Among VPSs, Aquasil exhibited the highest TSb value (5.1 MPa) compared to all other materials tested, irrespective of the viscosity. Within the heavy bodies, Aquasil TSb was statistically comparable to Affinis (4.93 MPa) and Flexitime (4.91 MPa), while within the light bodies Aquasil (4.98 MPa) was comparable to Exa'lence (4.03 MPa) and Flexitime (3.02 MPa). Comparing the different viscosities of products from the same manufacturer, heavy-body materials showed higher tensile strength values than the light bodies; such a difference proved to be not statistically significant (*P* > 0.05) for Aquasil (HB: 5.1 MPa, LB: 4.98 MPa—*P* = 1.000) and Impregum (HB: 1.49 MPa, LB: 1.47 MPa—*P* = 1.000). A different behavior was recorded for the VPES material, whose medium and light viscosities (MB = 3.16 MPa; LB = 4.03 MPa) showed significantly increased TSb values (*P* < 0.001) compared to the HB viscosity (1.42 MPa).

As for the TSb, also regarding the yield strength (YS), the HB VPSs showed slightly increased mean values compared to the corresponding LB viscosities; however the differences in yield strength were statistically significant just for Affinis (HB: 2.85 MPa, LB: 1.12 MPa).

Concerning the ultimate strain at break (USb), statistical analysis showed that heavy-body VPSs had significantly reduced mean values compared to the corresponding light bodies, with the exception of Affinis (HB: 47.19 mm, LB: 47.55 mm) and Aquasil (HB: 34.53 mm, LB: 45.88 mm). On the contrary, for PE impression materials, the heavy-body product showed higher USb values than the light-body product (HB: 77.17 mm, LB: 45.68 mm). The medium-body materials of some brands (Flexitime and Hydrorise) presented intermediate USb values, while for other brands the USb of the medium body was comparable to the corresponding LB (Aquasil) or HB (Exa'lence, Affinis). Comparing the different brands within the same viscosity, Heraeus Kulzer Flexitime and Zhermack Hydrorise showed the highest USb values both among the light bodies (101.25 mm and 90.39 mm, resp.) and among the medium bodies (81.59 mm and 77.61 mm, resp.). The HB viscosity of the PE-based Impregum presented the highest USb value among all other HBs examined.

Regarding the strain at yield point (Sy) the lowest values were generally recorded on HB viscosities. HB VPSs had steadily lower Sy values compared to LB viscosities. Only for PE impression materials the heavy body showed higher Sy values than the light body (HB: 48.23 mm, LB: 18.56 mm). Flexitime showed the highest Sy values among the LB products (56.57 mm) and the lowest values among the HB materials (9.43 mm).

## 4. Discussion

When an impression is removed from the mouth, the material must withstand the forces associated with separating the impression from the tooth and its surrounding tissues. The material located at or close to undercut areas could permanently deform on removal. Thus, elastic recovery is important in determining the accuracy of an impression material [9]. The clinical tear performance of a material also appears crucial [15, 16]: it involves complex interactions between polymer and fillers, flow to a particular film thickness, release properties from tooth and soft tissue, presence of internal and surface defects, and rate of impression withdrawal. Because of the complexities of integrating and measuring these properties, laboratory tests evaluating the propagation energy of a tear have been employed as common ways to evaluate elastic dental materials [9, 17]. New “hydrophilic” elastomeric impression materials have been recently introduced with the goals of reducing marginal voids and distortion in the impressions and improving the quality of gypsum dies, but there are still insufficient data on their mechanical properties.

Based on the results of the present study, the null hypothesis that there was no difference in the tensile properties among the different impression materials and consistencies tested was rejected.

Tensile strength at break is the maximum tensile stress applied in stretching a specimen to rupture [9]. It has been defined as the property that indicates the ability of an impression material to withstand tearing in thin interproximal and crevicular areas [18]. Statistical analysis showed significant differences in the tensile strengths of different products, supporting findings in the literature [9, 19]. VPSs consistently showed higher tensile strength values than PEs. Comparing the different viscosities of materials produced by the same manufacturer, heavy-body VPSs showed higher tensile strength values than the light bodies. It should however be considered that although heavy-body impression materials showed higher tensile resistance, the tensile properties of light bodies appear more clinically relevant, since the most likely torn portions of the impression are the thin interproximal and crevicular areas. This may highlight an advantageous peculiarity of the new VPES hybrid material, which showed very high tensile strength values for the light- and medium-body viscosities. The actual behavior of the MB viscosities proved to be quite unpredictable: despite what one could expect, only for Exa'lence and Affinis, the TSb of the medium body showed an intermediate TSb value between those of the corresponding HB and LB viscosities.

The yield strength determines the materials' ability to withstand stress without permanent deformation. The strain at yield point indicates the amount of undercut an impression material can overcome without permanent elastic deformation. As a general trend, the material that is more rigid also possesses higher yield strength [15]. Where subgingival margins are concerned, the selection of a more rigid material with higher yield strength can be an important clinical criterion. Walker at al. [20] recently suggested that high impression material rigidity and hardness are not predictors of impression removal difficulty. A performing material should display high yield strength and adequate elastic recovery and should require the expenditure of large amounts of energy to initiate and propagate tearing. The polymerized material have to maintain its elasticity under stresses created, for instance, when it flexes over tissue undercuts. The distortion of an impression material beyond its elastic range may cause permanent deformation and renders it inaccurate [15]. Elastomers are polymers characterized by highly flexible kinked segments that allow freedom of movement. Under stress (load/area), the segments will uncoil. Upon removal of the stress, an ideal elastomer will exhibit complete elastic recovery; the segments spring back to prestressed conformations and the piece returns to its original dimensions. Permanent deformation occurs upon elongation of the segments past the point where elastic recovery is possible. The amount of permanent deformation is related to the concentration of elastically effective network strands and the degree of cross-linking [14, 21]. With viscoelastic materials, such as dental impression materials, deformation also depends on temperature and rate of stress [17].

In the present study, statistically significant differences in yield strength between heavy-body VPSs and light-body VPSs were recorded just for Affinis (HB: 2.85 MPa, LB: 1.12 MPa). However, we found high coefficient of variations (standard deviation values) for both the yield strength and the strain at yield parameters; such wide variability might influence the statistical significance of results that should be interpreted with caution. An explanation for the recorded high standard deviations might be found in the testing apparatus: during tensile loading of samples, when testing tear energy, the tear could deviate from the central axis of the specimen and the observed elongation might not be accurate. Beside Affinis group, all the other tested VPSs showed slightly increased but statistically similar YS values when the HBs were compared with the corresponding LBs produced by the same manufacturer. This may probably suggest a weak influence of the different viscosities (HB, MB, and LB) on the YS of VPSs, in contrast with what had been observed for the TSb. On the other hand, the new VPES hybrid material yielded the highest YS with the LB viscosity. PE impression materials showed the lowest yield strength, regardless of the viscosity. This appears in accordance with Lu et al.'s work [9] that demonstrated that the PE impression materials tested had significantly lower tensile strength and higher strain in compression compared to new addition silicone materials.

From the standpoint of clinical application, materials with high tensile strength are not necessarily considered to be superior to the materials with low tensile strength. Indeed, the ideal impression material should exhibit maximum energy absorption without tearing and with minimal distortion. Nevertheless, according to the authors, it is also desirable that the material tears rather than deforms on a critical point such as a margin, as in clinical practice it appears easier to properly judge and consequently discard a torn impression, rather than a deformed one. As a consequence, an impression material showing a TSb/YS ratio sufficiently close to 1 (i.e., yield strength value relatively close to the corresponding tensile strength at break) should probably be preferred, especially concerning the light bodies, which are generally employed in thin interproximal and crevicular areas. From the present results, the TSb/YS ratios ranged between 0.396 (Acqu-LB group) and 0.764 (Hydro-MB group).

Among the light bodies investigated in the present study, both Acqu-LB and Exa-LB showed relatively high mean values for both tensile and yield strength. While for Exa-LB the TSb/YS ratio was relatively high (0.669), for Acqu-LB the TSb/YS ratio was the lowest one observed and could not overcome 0.396. This can be probably seen as a further clinical advantage of the new VPES hybrid material.

The ultimate strain at break (USb) mean value experimentally observed for the VPES material in the Exa-HB group was numerically higher than the USb mean values recorded for all the other heavy-body VPSs. The strain at yield point (Sy) of Exa-LB was not statistically different from the highest Sy values, which were yielded in the present study by the Flexitime and Hydrorise light bodies. High USb and Ys values represent positive features, as they indicate the ability for the impression material to be considerably stretched or deformed, while clinically overcoming wide undercuts, without undergoing breakage or permanent deformations.

Many studies [9, 14, 15, 20, 22–24] on tear strength have been carried out so far; however, standardized test methods have not been established. As a result, comparison between different impression materials using the available literature data still appears quite difficult [15, 17].

The ANSI/ADA standard specifies that tear strength specimens of dental elastomeric impression materials should be tested 1 hour following polymerization [25]. Nevertheless, impressions are clinically subjected to tensile forces immediately after the manufacturer's setting time. A previous research revealed that there are significant differences between testing immediately after the setting time and 24 hours following the setting time [23]. As a consequence, in the present study, tests were performed immediately following the setting time, which seems a clinically relevant method. Among the limitations of our investigation there is its specific in vitro nature; moreover, the elastic recovery from compressive and tensile strains was not considered. In addition, the applied ANSI/ADA specification just takes into consideration a thickness of 2 mm for the fabrication of samples. Future researches are necessary to simulate the clinical oral behavior of impression materials.

## 5. Conclusions

The choice of an impression material for particular applications should be based on the specific property data rather than on the type and class of the elastomer. With regard to the mechanical properties tested, considering all different viscosities, VPSs and VPESs showed higher in vitro results for tensile strength at break (TSb) and yield strength (YS) than PE. Heavy-body VPSs generally showed higher TSb values than the light bodies from the same manufacturer. Within the light bodies (LB) that are typically employed in the interproximal/subgingival areas and thus appear more subject to clinical tearing, the best performances in terms of TSb and YS were observed in the Acqu-LB and Exa-LB groups, with Exa-LB conveniently showing a relatively high TSb/YS ratio.

## Figures and Tables

**Table 1:** Information on the materials tested.

Group	Material (trade name)	Manufacturer	Viscosity	Composition	Setting time
Acqu-HB	Aquasil ULTRA DECA Heavy (dynamic mix)	Dentsply DeTrey GmbH, Konstanz, Germany	HB	Polyvinylsiloxane	Regular
Hydro-HB	Hydrorise Heavy (dynamic mix)	Zhermack SpA, Badia Polesine (RO), Italy	HB	Polyvinylsiloxane	Regular
Affi-HB	Affinis System Heavy Body 360 (dynamic mix)	Coltène/Whaledent AG, Altstätten, Switzerland	HB	Polyvinylsiloxane	Regular
Flexi-HB	Flexitime DYNAMIX Heavy Tray (dynamic mix)	Heraeus Kulzer GmbH, Hanau, Germany	HB	Polyvinylsiloxane	Regular
Impr-HB	Impregum PENTA Duosoft H (dynamic mix)	3M ESPE, Seefeld, Germany	HB	Polyether	Regular
Exa-HB	Exa'lence Regular Set Heavy (dynamic mix)	GC Corporation, Tokyo, Japan	HB	Vinylpolyether silicone	Regular

Acqu-MB	Aquasil ULTRA MONO (syringe automix)	Dentsply DeTrey GmbH, Konstanz, Germany	MB	Polyvinylsiloxane	Regular
Hydro-MB	Hydrorise Regular Body (syringe automix)	Zhermack SpA, Badia Polesine (RO), Italy	MB	Polyvinylsiloxane	Regular
Affi-MB	Affinis Regular Body (syringe automix)	Coltène/Whaledent AG, Altstätten, Switzerland	MB	Polyvinylsiloxane	Regular
Flexi-MB	Flexitime Medium Flow (syringe automix)	Heraeus Kulzer GmbH, Hanau, Germany	MB	Polyvinylsiloxane	Regular
Exa-MB	Exa'lence Medium Body (syringe automix)	GC Corporation, Tokyo, Japan	MB	Vinylpolyether silicone	Regular

Acqu-LB	Aquasil ULTRA LV (syringe automix)	Dentsply DeTrey GmbH, Konstanz, Germany	LB	Polyvinylsiloxane	Regular
Hydro-LB	Hydrorise Light Body (syringe automix)	Zhermack SpA, Badia Polesine (RO), Italy	LB	Polyvinylsiloxane	Regular
Affi-LB	Affinis Light Body (syringe automix)	Coltène/Whaledent AG, Altstätten, Switzerland	LB	Polyvinylsiloxane	Regular
Flexi-LB	Flexitime Light Flow (syringe automix)	Heraeus Kulzer GmbH, Hanau, Germany	LB	Polyvinylsiloxane	Regular
Impr-LB	Impregum Garant Duosoft L (syringe automix)	3M ESPE, Seefeld, Germany	LB	Polyether	Regular
Exa-LB	Exa'lence Light Body (syringe automix)	GC Corporation, Tokyo, Japan	LB	Vinylpolyether silicone	Regular

HB = heavy body; MB = medium body; and LB = light body.

**Table 2:** Dimensions for dumbbell test specimens according to type 1 of the ISO 37:2005 specifications and/or type C of ASTM.D412 specifications. Each uppercase letter relates to the corresponding dimension as indicated in Figure 1.

Dimension (mm)	
*A*: overall length (minimum)	115
*B*: width of ends	25.0 ± 1
*C*: length of narrow portion	33 ± 2
*D*: width of narrow portion	6 ± 0.4
*E*: transition radius outside	14 ± 1
*F*: transition radius inside	25 ± 2
*G*: thickness of narrow portion	2 ± 0.2
*H*: test length	25 ± 0.5

**Table 3:** Mean values (and standard deviations, SD) recorded for tensile strength at break (TSb), yield strength (YS), ultimate strain at break (USb), and strain at yield point (Sy) in the different experimental groups.

TSb (SD) (MPa)		YS (SD) (MPa)	
Acqu-HB^*∗*^	5.10^a^ (0.40)	Affi-HB^*∗*^	2.85^a^ (1.26)
Acqu-LB^*∗∗∗*^	4.98^a,b^ (0.39)	Acqu-MB^*∗∗*^	2.70^a,b^ (1.28)
Flexi-HB^*∗*^	4.90^a,b^ (0.99)	Exa-LB^*∗∗∗*^	2.70^a,b^ (0.91)
Affi-HB^*∗*^	4.88^a,b^ (0.52)	Acqu-HB^*∗*^	2.36^a,b,c^ (0.97)
Acqu-MB^*∗∗*^	4.18^b,c^ (0.59)	Flexi-HB^*∗*^	2.12^a,b,c,d^ (0.99)
Exa-LB^*∗∗∗*^	4.03^c^ (0.56)	Acqu-LB^*∗∗∗*^	1.97^a,b,c,d^ (0.81)
Hydro-HB^*∗*^	3.19^d^ (1.02)	Flexi-LB^*∗∗∗*^	1.78^a,b,c,d^ (1.01)
Exa-MB^*∗∗*^	3.16^d^ (0.52)	Affi-MB^*∗∗*^	1.77^a,b,c,d^ (0.64)
Flexi-LB^*∗∗∗*^	2.93^d,e^ (0.54)	Hydro-HB^*∗*^	1.60^b,c,d^ (0.98)
Affi-MB^*∗∗*^	2.60^d,e,f^ (0.37)	Flexi-MB^*∗∗*^	1.47^b,c,d^ (0.56)
Hydro-LB^*∗∗∗*^	2.30^e,f,g^ (0.31)	Hydro-MB^*∗∗*^	1.38^c,d^ (0.46)
Flexi-MB^*∗∗*^	2.10^f,g,h^ (0.34)	Hydro-LB^*∗∗∗*^	1.35^c,d^ (0.47)
Affi-LB^*∗∗∗*^	2.03^f,g,h^ (0.55)	Exa-MB^*∗∗*^	1.35^c,d^ (0.69)
Hydro-MB^*∗∗*^	1.77^g,h^ (0.31)	Affi-LB^*∗∗∗*^	1.12^c,d^ (0.51)
Impr-LB^*∗∗∗*^	1.46^h^ (0.19)	Impr-HB^*∗*^	1.11^d^ (0.46)
Impr-HB^*∗*^	1.44^h^ (0.42)	Exa-HB^*∗*^	0.96^d^ (0.32)
Exa-HB^*∗*^	1.42^h^ (0.29)	Impr-LB^*∗∗∗*^	0.94^d^ (0.26)

USb (SD) (mm)		Sy (SD) (mm)	

Hydro-LB^*∗∗∗*^	101.26^a^ (13.86)	Flexi-LB^*∗∗∗*^	56.57^a^ (27.60)
Flexi-LB^*∗∗∗*^	90.39^a,b^ (15.72)	Hydro-LB^*∗∗∗*^	51.43^a,b^ (19.46)
Hydro-MB^*∗∗*^	81.59^a,b^ (21.25)	Flexi-MB^*∗∗*^	51.01^a,b^ (17.23)
Flexi-MB^*∗∗*^	77.61^b^ (9.71)	Impr-HB^*∗*^	48.23^a,b,c^ (22.42)
Impr-HB^*∗*^	77.17^b^ (21.11)	Exa-LB^*∗∗∗*^	45.74^a,b,c^ (15.06)
Exa-LB^*∗∗∗*^	71.69^b,c^ (12.95)	Hydro-MB^*∗∗*^	35.61^a,b,c,d^ (29.68)
Exa-MB^*∗∗*^	56.56^c,d^ (11.17)	Affi-MB^*∗∗*^	35.09^a,b,c,d^ (13.29)
Affi-MB^*∗∗*^	56.43^c,d^ (12.52)	Aqua-MB^*∗∗*^	28.32^b,c,d,e^ (12.86)
Exa-HB^*∗*^	54.21^c,d,e^ (17.76)	Affi-LB^*∗∗∗*^	27.23^b,c,d,e^ (15.44)
Affi-LB^*∗∗∗*^	47.55^b,c,d,e^ (10.21)	Affi-HB^*∗*^	26.24^c,d,e^(12.18)
Affi-HB^*∗*^	47.19^d,e^ (6.01)	Aqua-LB^*∗∗∗*^	18.74^d,e^ (5.67)
Aqua-MB^*∗∗*^	46.09^d,e^ (8.08)	Impr-LB^*∗∗∗*^	18.56^d,e^ (6.07)
Aqua-LB^*∗∗∗*^	45.88^d,e,f^ (5.07)	Exa-MB^*∗∗*^	16.92^d,e^ (13.27)
Impr-LB^*∗∗∗*^	45.68^d,e,f^ (10.81)	Aqua-HB^*∗*^	15.64^d,e^ (6.29)
Hydro-HB^*∗*^	41.77^d,e,f^ (14.53)	Exa-HB^*∗*^	14.01^d,e^ (10.37)
Aqua-HB^*∗*^	34.53^e,f^ (3.65)	Hydro-HB^*∗*^	12.09^d,e^ (8.42)
Flexi-HB^*∗*^	26.23^f^ (2.80)	Flexi-HB^*∗*^	9.43^e^ (4.29)

The same superscript letters indicate no statistically significant differences (*P* > 0.05).

^*∗*^Heavy body.

^*∗∗*^Medium body.

^*∗∗∗*^Light body.
